# Genome-Wide Scan for Bats and Dolphin to Detect Their Genetic Basis for New Locomotive Styles

**DOI:** 10.1371/journal.pone.0046455

**Published:** 2012-11-06

**Authors:** Yong-Yi Shen, Wei-Ping Zhou, Tai-Cheng Zhou, Yan-Ni Zeng, Gui-Mei Li, David M. Irwin, Ya-Ping Zhang

**Affiliations:** 1 State Key Laboratory of Genetic Resources and Evolution, Kunming Institute of Zoology, the Chinese Academy of Sciences, Kunming, China; 2 Department of Molecular and Cell Biology, School of Life Sciences, University of Science and Technology of China, Hefei, Anhui, China; 3 Laboratory for Conservation and Utilization of Bio-resources, Yunnan University, Kunming, China; 4 School of Life Science, East China Normal University, Shanghai, China; 5 Department of Laboratory Medicine and Pathobiology, University of Toronto, Toronto, Ontario, Canada; 6 Banting and Best Diabetes Centre, University of Toronto, Toronto, Ontario, Canada; CSIRO, Australia

## Abstract

For most mammals, running is their major locomotive style, however, cetaceans and bats are two mammalian groups that have independently developed new locomotive styles (swimming and flying) from their terrestrial ancestors. In this study, we used a genome-wide comparative analysis in an attempt to identify the selective imprint of the development of new locomotive styles by cetaceans and bats to adapt to their new ecological niches. We found that an elevated proportion of mitochondrion-associated genes show evidence of adaptive evolution in cetaceans and on the common ancestral lineage leading to bats, compared to other terrestrial mammals. This result is consistent with the fact that during the independent developments of swimming and flying in these two groups, the changes of energy metabolism ratios would be among the most important factors to overcome elevated energy demands. Furthermore, genes that show evidence of sequence convergence or parallel evolution in these two lineages were overrepresented in the categories of energy metabolism, muscle contraction, heart, and glucose metabolism, genes that perform functions which are essential for locomotion. In conclusion, our analyses showed that on the dolphin and bat lineages, genes associated with locomotion not only both show a greater propensity to adaptively evolve, but also show evidence of sequence convergence, which likely reflects a response to a common requirement during their development of these two drastic locomotive styles.

## Introduction

Most mammals are terrestrial and their major locomotive style is walking/running. Different types of locomotion call for very different physiological and anatomical adaptations such as bone structures, body shape, and energy metabolism. Changes in locomotive styles require a large number of physiological and morphological changes, yet during the evolutionary history of mammals, cetaceans and bats have independently evolved dramatically new locomotive styles – swimming and flying.

The highest maximal aerobic power in mammals have been reported for flying bats [1], which have peak oxygen consumptions 2.5–3 times greater than those for running mammals with similar body mass [1], [2]. Bat pectoralis muscle had high myofibrillar ATPase activities and fast contractile properties [3]. The bat's heart is three times as large as that of a terrestrial mammal of comparable size to pump around the oxygen required for sustained flight [4].

Cetaceans are descendants of terrestrial mammals that entered the water environment roughly 65 million years ago [5], [6]. The structural and functional adaptations, which enable them to cope with an underwater lifestyle, involve intricate adjustments in most organs. Water is 800 times denser and 60 times more viscous than air [7], thus this environment has a much higher resistance during locomotion. Therefore, the energy costs of swimming are much higher than for running. Especially, their semi-aquatic ancestral marine mammal species likely incurred costs of energy that were 2.4 to 5.1 times higher than those of running [8]. Thus, the energy barrier must have played a major role in the evolution of this group [9]. Changes in their skeletal musculature also are bound to be important [10].

Although bats and cetaceans have very different lifestyles (flying in the air and swimming in the water), both represent the development of new locomotive styles from terrestrial ancestors to fit for the new ecological niches. During the evolution of these new locomotive styles, a series of changes must have occurred to multiple genes to allow the necessary morphological and physiological adaptations. Natural selection should leave an imprint on these genes, therefore, in this study, we conducted genome-wide scans on the available genome sequences (which includes two bats and one dolphin) to identify genes showing evidence of adaptive evolution and convergent evolution on the bat and cetacean lineages during the evolution of flying and swimming ability from terrestrial ancestors.

## Results and Discussion

**Figure 1:**
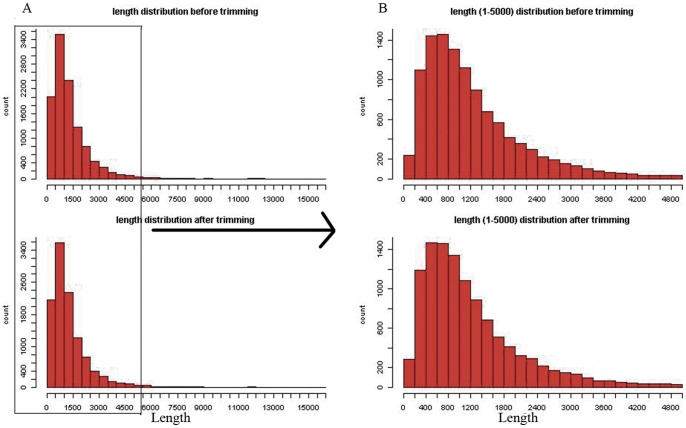
The length distribution of orthologous genes before and after trimming. (A) The length distribution of all of the orhtologous genes; (B) The length distribution of orthologous genes with length from 1 to 5,000.

Bats and cetaceans represent two groups of mammals that have independently evolved new locomotive styles. To investigate the genetic basis for the development of these new locomotive styles, we used the available genomes for genome-wide scan. Single-copy orthologs from all seven genomes (human, mouse, horse, cow, dolphin, megabat, and microbat), were identified, aligned, and trimmed for quality control (details in Materials and Methods). A total of 11,268 orthologous genes, the gene lengths ranging from 102 to 15978 bp, were collected. The distribution of the lengths of the gene sequences before and after trimming is shown in Figure 1. After trimming, the lengths of the genes were shorter, however the shape of their distributions is similar with that before trimming. Thus, this treatment did not lose too much sequence information.

**Figure 2:**
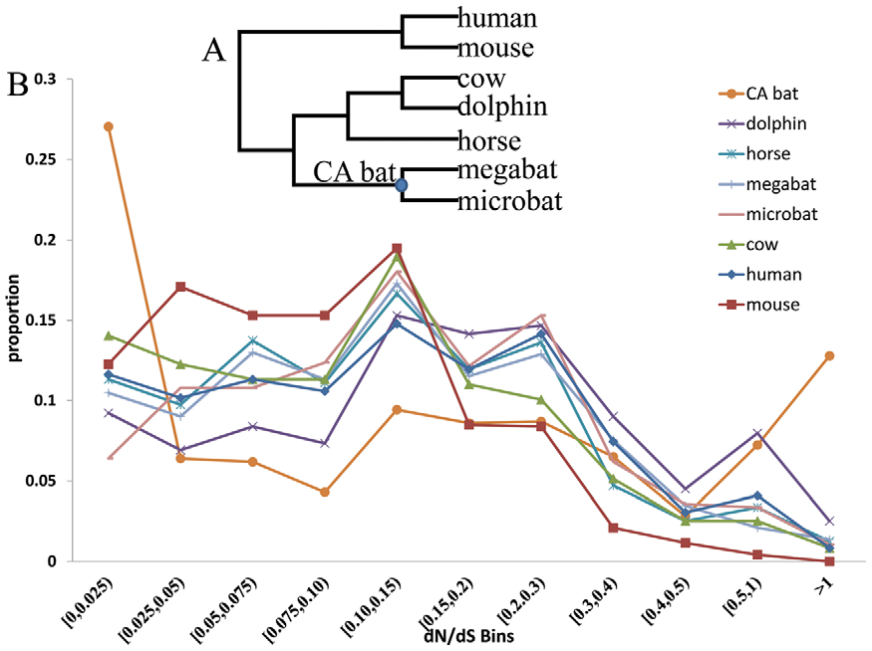
The analyses of selective pressures on energy metabolism genes. (A) Species tree that was used as the guide tree for the selection analyses. CA bat is the common ancestor of bats; (B) Proportion of energy metabolism genes with given *dN/dS* ratios for lineages leading to eight mammals and the common ancestor of bats (CA bat).

A gene-level approach, based on the ratio of nonsynonymous (*Ka* or *dN*) to synonymous (*Ks* or *dS*) substitution rates (ω =  *Ka/Ks* or *dN/dS*), was then used to identify lineages showing evidence for positive selection, using the CODEML algorithm from the PAML 4 package [11]. The free-ratio model (M1 model) was used to obtain parameters (including *dN*, *dS*, *dN/dS*, N*dN, and S*dS values) for all of the genes on each of the branches, using the accepted mammalian species tree (Figure 2A) [6], [12] as a guide tree. Parameters (*dN*, *dS*, *dN/dS*, N*dN, and S*dS values) were obtained for eight lineages in the tree: CA bat (Common Ancestor of bats), dolphin, human, mouse, cow, horse, megabat, and microbat. Genes were discarded if they had any one of the following values: *dS*>1, N greater than the RefSeq length, *N*+*S*>RefSeq length by 50 or more bp, according a previous study [13].

Genes in the ω>1 bin are very likely to have experienced positively selection, thus, we used DAVID [14], [15] to do a functional annotation clustering of the genes with ω>1 for each species. This results of the clustering of potentially adaptively evolving genes on the CA bat lineage showed an overrepresentation of the following GO gene terms: GO:0005739∼mitochondrion (*P* = 0.036), GO:0006979∼response to oxidative stress (*P* = 0.047), GO:0001666∼response to hypoxia (*P* = 0.041), GO:0045822∼negative regulation of heart contraction (*P* = 0.034) (details in Table S1). The GO term GO:0005739∼mitochondrion was not significantly overrepresented in any of the other terrestrial mammals (human, mouse, cow and horse; *P* values range from 0.39 to 1). The significant overrepresentation of the GO gene term mitochondrion in bats agrees with a previous study that showed that adaptive evolution had occurred on genes involved in energy metabolism (mitochondrial genes and nuclear-encoded mitochondrial proteins genes) during the attainment of flight by bats [16]. Results of the analysis for the dolphin lineage showed that the adaptively evolving genes were mainly overrepresented for GO gene terms associated with immune system (details in Table S2). This may reflect the great change in the environment of these species, moving from land to an aquatic environment with very different pathogens.

Energy metabolism is very different in flying and diving mammals than their terrestrial ancestors [1], [2], [9]. And ATP (adenosine triphosphate) that is generated by cells for locomotion is mainly produced by aerobic respiration in the mitochondria. To provide a more refined view of the pattern of the selective pressures on energy metabolism genes, we sorted genes of the GO term GO:0005739∼mitochondrion into 11 non-overlapping bins according to their *dN/dS* values: [0, 0.025); [0.025, 0.05); [0.05, 0.075); [0.075, 0.1); [0.1, 0.15); [0.15, 0.2); [0.2, 0.3); [0.3, 0.4); [0.4, 0.5); [0.5, 1]; >1. The proportions of energy metabolism genes in each of these *dN/dS* bins for each of the eight lineages are shown in Figure 2B. The *dN/dS* bin with ω>1, contains 12.79% and 2.52% of the genes for the CA bat and dolphin, respectively, proportions that were much higher than that observed for the mouse, 0%, cow, 0.84%, horse, 1.26%, megabat, 1.36%, microbat, 1.05%, or human, 0.84% (Fig. 2B). As indicated above, genes in the ω>1 bin very likely experienced positive selection. This observation suggests that on the common ancestor leading to bats a high proportion of energy metabolism genes underwent positive selection to adapt for the energy-demanding activity – flying. The dolphin lineage has the second highest proportion of mitochondrion genes to show evidence of positive selection during its evolution and adaptation to swimming. Other terrestrial mammals have very low proportions of mitochondrion genes suggesting evidence of positive selection, ranging from 0 to 1.26%, suggesting little adaptation is associated with the retained locomotive style. The ability to fly was attained on the CA bat lineage, thus for the two bat lineages, the locomotive style did not change. In agreement with this, the proportions of mitochondrion genes suggesting evidence of positive selection on these two lineages are also low, being 1.36% and 1.05%, values that are similar to those of terrestrial mammals (Figure 2B). Thus the adaptive evolution of the mitochondrion genes only occurred on the CA bat lineage and has now been replaced largely by purifying selection within bats. In conclusion, the CA bat and dolphin lineages – two lineages that have evolved new locomotive styles, both have a high proportion of their energy metabolism genes in the ω>1 bin, suggesting evidence of adaptive evolution (Figure 2B).

The results of this analysis showed that the two independent developments of new locomotive styles by bats and cetaceans, despite requiring different types of physiological and morphological changes, both may involve adaptive evolution of energy metabolism (mitochondria) genes. This observation is consistent with the fact that the different locomotive styles consume differing amounts of energy, with swimming and flying requiring more than running, and thus during the evolution of the new locomotive styles, changes in energy metabolism represents a great barrier. While some studies suggest that the total energetic cost of flight and swimming are more optimal than running [8], the maximum energy metabolic rate for bats is high [17]–[19], and the semi-aquatic lifestyle of Cetaceans, which occurred during the early evolution of this group, is very energy-consuming [8]. Especially, Cetaceans have to provide enough energy in hypoxia when diving, this would restrict their energy metabolism system [20].

Despite the free ratio model, we also used branch-site model to further detect the selection pressure. We found that positive selection genes on the CA bat lineage were significantly overrepresented in gene terms that associated with muscle and glucose transmembrane transportation (details in Table S3). While on the dolphin lineage, positive selection genes were significantly overrepresented in gene terms that associated with muscle, eyes and ATPase (details in Table S4). Significant enrichments of muscle genes in both CA bat and dolphin lineages suggest that the two independent developments of new locomotive styles by bats and cetaceans, both required muscle changes to fit for the new drastic locomotive styles. Results of the overrepresentation of positive selective genes for GO gene terms associated with eyes on the dolphin lineage may reflect the great change in the light environment of cetaceans, moving from land to an aquatic environment.

Selection pressure only tells part of the story of evolution. In addition to examining the pattern of selection pressure, we also determined whether genes evolved convergently or in parallel on the dolphin and CA bat lineages. Possible instances of convergent or parallel sequence evolution were detected in 8.58% of the genes in the orthologous gene set on the CA bat and dolphin lineages. A total of 967 of the 11,268 (8.58%) of the seven mammalian genome orthologous gene set have at least one convergent/parallel amino acid change on the CA bat and dolphin lineages. Genes showing convergent or parallel evolution were overrepresented, according to DAVID [14], [15], for the following GO terms: GO:0016887∼ATPase activity (*P* = 0.003), GO:0006006∼glucose metabolic process (*P* = 0.017), GO:0007507∼heart development (*P* = 0.008), and GO:0006936∼muscle contraction (*P* = 0.030) (details in Table S5). In addition to changes in energy metabolism, other structural modifications were required to allow flying and swimming to become possible, including the development of powerful muscles and heart, and an efficient oxygen absorption and transport system [21]–[23]. Sequence convergence in these gene terms likely reflect common physiological changes needed for the independent development of these two new locomotive styles, despite flying and swimming being very different.

For this analysis we used two draft genome sequences, those for the dolphin and megabat, and these may have limited our ability to identify convergent genes. To test the reliability of our methods at finding genes undergoing convergent evolution, we chose the dolphin branch and microbat branch in Figure 2A, and evaluated the extent of convergent evolution using the same methods described above. We detected parallel amino acid changes in the *Prestin* gene. The *Prestin* gene has been demonstrated to have evolved convergently between echolocating bats and dolphins in response to their need to detect high frequency sounds [24], [25].

Cetaceans and bats have independently evolved dramatically new locomotive styles – swimming and flying. Flight and swimming have enabled bats and cetaceans to exploit a variety of foraging niches inaccessible to other mammals. Many physiological and structural modifications were needed for these locomotive style changes. In this study, we demonstrate that during the emergence of bats and cetaceans from their ancestors, at least some of their locomotion associated genes (energy metabolism, muscle and heart genes) have independently undergone adaptive evolution, with quite a few undergoing convergent evolution. Our results reveal convergent evolutionary patterns in locomotion associated genes on the lineages leading to dolphin and bat, which likely reflect shared demands for elevated levels of energy, and changes to muscle and heart required for their new locomotive styles. Despite the convergent evolutionary patterns in these locomotion-associated genes, we found they showed very different selective pressure in other gene terms, which may reflect their adaption to very different ecological niches.

## Materials and Methods

### Source of data and primary treatments

Genomes from seven mammals, including human (*Homo sapiens*, GRCh37.64), mouse (*Mus musculus*, NCBIM37.64), horse (*Equus caballus*, EquCab2.64, 6.79X), cow (*Bos taurus*, UMD3.1), dolphin (*Tursiops truncatus*, turTru1.64, 2.59X), megabat (*Pteropus vampyrus*, pteVam1.64, 2.63X), and microbat (*Myotis lucifugus*, Myoluc2.0, 7X), were selected for this study, which included two bats and one dolphin. Orthologous genes from the seven genomes were identified using the Ensembl ortholog_one2one gene database (version 64, September 2011) [26] for each pair of genomes. Only genes that were one-to-one orthologs for every pair of genomes for the seven species were used in this analysis. If genes had more than one transcript, we aligned the longest transcript pairs for all seven species with the Kalign program [27]. The genomes of two species, megabat and dolphin, are of only draft quality raising the possibility that sequencing or assembly errors may interfere with the ability to identify genes that have experienced positive selection. To reduce the rate of false positive prediction, we used a series of analyses developed in a previous study [16]. Briefly, as a first step we deleted all gaps and “N” from the alignments. Next, to identify sequencing errors, incorrect alignments or nonorthologus regions in the alignments, we used a 15 bp sliding window on each alignment, moving the sliding window by one codon for each step to the end of the alignment, and for each window calculated the lowest similarity of an alignment pair of the seven species within the sliding window. Alignment regions with a lowest similarity of <7/15 were discarded, as these may have included errors in the sequence or assembly. After the deletion step, if the remaining alignment was shorter than 100 bp, then the entire alignment was discarded. Our final data set contained 11,268 genes.

### Selection analyses

Alignments and consensus trees were used for posterior molecular evolutionary analysis. We used a gene-level approach based on the ratio (ω) of nonsynonymous (*Ka* or *dN*) to synonymous (*Ks* or *dS*) substitutions rate (ω = *Ka/Ks* or *dN/dS*) to identify potential positive selection, using the CODEML algorithm from the PAML 4 package [11]. We used the free-ratio model to estimate parameters (such as *dN*, *dS*, *dN/dS* values) for the genes of each branch of the tree. Then, branch-site model was used to further detect the positive selection signals. Test 1 (branch-site model vs. site model M1a) and test 2 (branch-site model vs. branch-site model with fixed ω_1_ = 1) [28] were conducted to control the false positive signals.

### Functional annotation clustering

Human NCBI EntrezGene IDs were used in all of the gene ontology analyses. Orthologs of the human genes were retrieved from Ensembl Biomart. For uniformity of functional annotation enrichment results, we used the human NCBI EntrezGene IDs to refer both to the human genes and to their putative orthologs. We sorted genes of the GO term GO:0005739∼mitochondrion into 11 non-overlapping bins according to their *dN/dS* values: [0, 0.025); [0.025, 0.05); [0.05, 0.075); [0.075, 0.1); [0.1, 0.15); [0.15, 0.2); [0.2, 0.3); [0.3, 0.4); [0.4, 0.5); [0.5, 1]; >1. We used DAVID [14], [15] as a functional annotation clustering tool for each “species and *dN/dS* bin” combination to group genes with shared annotations. The algorithm assigns a significance *P* value, corrected for multiple testing, to each subgroup representing a gene ontology annotation within the cluster and an enrichment score to the whole cluster. The clusters with the higher enrichment scores consisted of subgroups with the higher significance values, and thus these clusters provided an integrated view of the more significantly enriched or overrepresented gene functional categories within each *dN/dS* bin. The enrichment score for each annotation cluster was based on the geometric mean of the *P* values of the cluster’s assorted annotations. The options were set to their default values.

### Convergent evolution analyses

Convergent and parallel changes were identified using the PAML package [11] to reconstruct the most likely ancestral states, and then an in-house Perl program to count the number of convergent/parallel amino acid replacements for a specified pair of branches. If the posterior probability of the reconstructed ancestral amino acid site was less than 90%, then they were discarded, as their state was deemed unreliable. The statistical significance of these amino acid changes was tested with the method developed by Zhang and Kumar [29].

## Supporting Information

Table S1 **Functional annotation clustering for genes with ω>1 for CA bat lineage.**
(XLS)

Table S2 **Functional annotation clustering for genes with ω>1 for dolphin lineage.**
(XLS)

Table S3 **Functional annotation clustering for positive selection genes based on branch-site model for CA bat lineage.**
(XLS)

Table S4 **Functional annotation clustering for positive selection genes based on branch-site model for dolphin lineage.**
(XLS)

Table S5 **Functional annotation clustering for genes that have evidence of convergent or parallel sequence evolution on the CA bat and dolphin lineages.**
(XLS)
